# Implicit effect of visual long-term memory for nonverbal objects on recognition judgment

**DOI:** 10.3758/s13414-025-03108-4

**Published:** 2025-06-20

**Authors:** Tomoe Masuoka, Megumi Nishiyama, Yuna Tsurusaki, Takafumi Terasawa

**Affiliations:** 1 Faculty of Nursing, Japanese Red Cross Hiroshima College of Nursing, 1-2 Azinadai-Higashi, Hatsukaichi City, Hiroshima, 738-0052 Japan; 2 Department of Criminal Psychology, University of Human Environments, Aichi, Japan; 3 Kamogata Nishi Elementary school, Okayama, Japan; 4 Faculty of Education, Okayama University, Okayama, Japan

**Keywords:** Visual perception, Object recognition, Long-term memory

## Abstract

This study uses an indirect recognition procedure to examine whether prior exposure to nonverbal visual objects affects recognition judgments in later, unrelated recognition tests. We also examined the effect of matching operations between study and test on recognition judgments. The experiment consisted of two sessions. The first session was an incidental learning task: Each object was presented twice, and participants were asked to count the number of corners of the presented object. In the second session after 3 weeks, participants performed the same task as in the first session and then performed an unexpected recognition test. In this test, participants were asked to identify whether the presented object had appeared in the second session. To unify the operation between study and test, some participants were required to count the number of corners of the presented object before the recognition judgment. The results revealed that recognition performance for the objects that appeared in the first session was significantly different from that of objects that had not appeared, even when participants were not asked to recall the episode of the first session when performing the recognition test. Although the results of the effect of the matching operation suggested a negative effect on recognition, the results were unclear. This finding indicates that representations for nonverbal objects are preserved for at least 3 weeks. This also highlights the need to consider the implicit effect of a brief prior experience on recognition judgments.

Recent studies of visual memory have shown that precise representations of individual objects or scenes can be retained in visual long-term memory (Brady et al., 2008, 2009; Hollingworth, 2005; Hollingworth & Henderson, 2002; Konkle et al., 2010a, 2010b; Miner et al., 2020). For example, Brady et al. (2008) showed 2,500 real-world object pictures to participants for 3 s per item and then asked them to perform a two-alternative forced-choice test in which a studied item was discriminated from a nonstudied item. The percentage of correct answers on recognition was considerable, even when the studied and nonstudied items had high similarity in visual characteristics. These studies have shown that visual memory for objects or scenes have high fidelity, and moreover, that when the objects contain conceptual information, memory for them are better recalled than when the objects are nonconceptual (Brady et al., 2011, 2019; Konkle et al., 2010b; Wiseman & Neisser, 1974). Previous research on human memory theory has been in agreement, arguing that long-term memory is organized by semantic networks (Collins & Loftus, 1975; Collins & Quillian, 1969) and that deeper processing (elaboration that involves forming associations with preexisting knowledge) is necessary to store information in long-term memory (Craik & Lockhart, 1972; Craik & Watkins, 1973). However, the ability of human to encode and recall certain visual information is not solely dependent on whether that information have conceptual information or not. In fact, even when stimuli have poor conceptual information, their representations are retained in memory. For example, Brady et al. (2019) used images of unambiguous faces, ambiguous faces, and nonface images as stimuli and showed that the processing of stimuli as meaningful contributed to performance on the recognition task (i.e., highest recognition performance was observed when images were unambiguous faces). They also reported, however, that the representations were retained in long-term memory even when stimuli were nonfaces. Considering this, we aimed to examine long-term persistence of memory for nonconceptual visual information using novel objects and longer delay. If even stimuli are novel and contain poor conceptual information but long-term memory for that objects are observed, it would contribute to add experimental evidence for the long-term persistence of visual memory. Specifically, the present study detected that visual memory representations for nonverbal objects acquired in a prior experience can be retained for at least 3 weeks and influence subsequent recognition performance implicitly.

## Implicit effect of prior experience on recognition

Previous studies for the priming effect have showed that representations of nonverbal and novel visual stimuli that lack preexisting semantic knowledge are preserved in long-term memory (DeSchepper & Treisman, 1996; Gabrieli et al., 1990; Musen & Treisman, 1990). The priming effect is the unconscious influence of having previously processed a stimulus on the subsequent processing for that stimulus (Tulving & Schacter, 1990). For example, Musen and Treisman (1990) presented participants with visual patterns by connecting lines on a 3 × 3 dot matrix, for 3 s per item as an initial study task. After that, in a memory test, participants were randomly presented both studied and nonstudied patterns with brief masking and were asked to draw what they had seen on the display. The percentage of correct drawings for previously studied patterns was significantly higher than that for nonstudied patterns, even a week after the initial study task. DeSchepper and Treisman (1996) also reported similar results. Using a negative priming procedure, they showed that representations of shapes that were novel and abstract were stored involuntarily and preserved for up to a month. These findings suggest that memory representations for nonconceptual visual information are retained quite a long time implicitly. However, it may be difficult to observe long-term persistence of nonconceptual visual information in a recognition task. For instance, although Musen and Treisman (1990) demonstrated the long-term priming effect of novel visual patterns, they also found that recognition performance dropped over time when participants had to identify one pattern that appeared in the initial study task from a group of four patterns, with the remaining three being new and different ones. Recognition tasks have been originally used as a procedure to detect consciously recalled memory. Thus, when stimuli have poor conceptual information, those representations may be difficult to intentionally maintain and to recall (McKeown et al., 2020).

It should be noted, however, that recognition judgments are not only supported by memory that is recalled consciously but also by automatic memory processes based on past experiences. For example, several studies in recent years have reported that detailed memory representations of nonverbal visual stimuli formed in prior experience automatically influence subsequent recognition performance (Masuoka et al., 2018a, 2018b; McKeown et al., 2014, 2020; Nishiyama & Kawaguchi, 2014). McKeown et al. (2014) used nonverbal objects to prevent maintenance through rehearsal and verbal encoding and showed that detailed representations formed in a previous experience did not decay over time and were used automatically in a subsequent recognition task. They used the recent-probe task, where target objects were presented for a brief period and a probe object was presented after an interstimulus interval. Participants were asked to decide whether the probe matched one of the target objects. The probe object was presented according to three conditions: matched the target object presented in the current trial (positive probe), matched the target object presented in the previous trial (recent-negative probe), and a completely novel object (nonrecent-negative probe). The response times and accuracy of the recent-negative probe were significantly slower and lower, respectively, than those of the nonrecent-negative probe, confirming proactive interference on the recent-negative probe, caused by the representation acquired in the previous trial. This proactive interference is not reduced even if an interval of tens of seconds was given between the previous and current trial (McKeown et al., 2020). Their results are in agreement with prior studies of priming that have shown robust memory representations for nonverbal visual stimuli and, furthermore, show that the representations formed in the prior trial implicitly influence current recognition performance that is not directly related to them. It is well known that performances on a recognition task are better when stimuli are meaningful for participants (Brady et al., 2019; Konkle et al., 2010b). In addition to this, focusing on an implicit effect of prior experience on recognition, long-term effects of slight exposure of visual information that are difficult to consciously memorize may be confirmed, not only in the priming but also in a recognition judgment. Therefore, in the present study, using the indirect recognition procedure, a procedure to detect an implicit effect of prior experience on recognition (Terasawa & Ohta, 1993), we aimed to examine the effect of exposure with nonconceptual visual information 3 weeks earlier on subsequent recognition judgments. If results are obtained in which the effect of slight experience that is no longer directly related to objects for which a recognition judgment is required over long durations, this would be evidence that underscores an importance of experience in a recognition process.

## Indirect recognition procedure

**Fig. 1:**
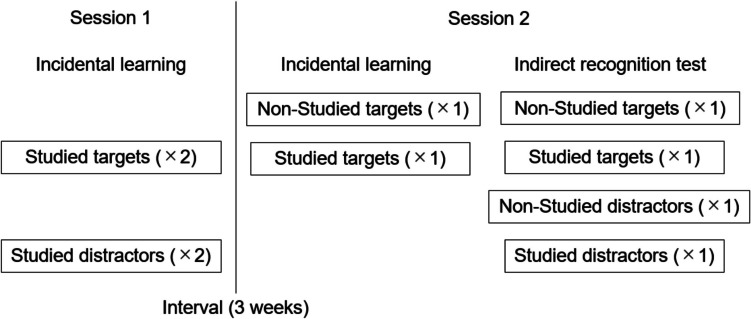
Structure of the indirect recognition procedure and object sets used for each task

The indirect recognition procedure is an experimental approach for detecting the automatic interference effect of prior experience on subsequent recognition performance. This procedure consists of two sessions (Fig. 1). The first session (Session 1) is an incidental learning task in which participants are asked to encode for the presented items while preventing intentional memorization. In this task, two types of items are presented: items that will be used as targets in a subsequent recognition test (“studied targets”) and items that will be used as distractors in that test (“studied distractors”). Following an interval, the second session (Session 2) is conducted. As in Session 1, in Session 2, an incidental learning task is carried out first. In this task, two types of items are presented. Both items will be used as targets in the subsequent recognition test, but one of them has already appeared in Session 1 (“studied targets”), and another is novel (“nonstudied targets”). Immediately afterwards, there is a surprise recognition test, referred to as the indirect recognition test, in which participants are asked to identify whether the presented item had appeared in the incidental learning task of Session 2. In this test, four types of items are presented: “studied targets” that appeared in the incidental learning task of Sessions 1 and 2, “nonstudied targets” that appeared only in the incidental learning task of Session 2, “studied distractors” that appeared in the incidental learning task of Session 1, and “nonstudied distractors” that are completely novel.

Since both “studied targets” and “nonstudied targets” were presented in the incidental learning task of Session 2, the correct response in the indirect recognition test to both of these types of target items was “Yes.” In this procedure, the rates of “Yes” responses to both the “studied targets” and the “nonstudied targets” are calculated as the respective hit rates. If the representations of “studied targets” formed in Session 1 were retained through the interval between Sessions 1 and 2 and affect recognition judgment, a difference should be observable in the hit rates for “studied targets” and “nonstudied targets.” Similarly, for the distractor items, both “studied distractors” and “nonstudied distractors” were not presented in the incidental learning task in Session 2. Thus, the rates of falsely responding to these items with a “Yes” are regarded as the false-alarm rates. The rate of “Yes” responses to both the “studied distractors” and the “nonstudied distractors” are calculated as the respective false-alarm rates. If the representations of “studied distractors” are retained after an interval and affect the recognition judgment, a difference should be observable in the false-alarm rates for “studied distractors” and “nonstudied distractors.” Thus, this procedure does not examine whether participants correctly discriminate whether or not the items had appeared in the incidental learning task of Session 2. Instead, it examines the effects of exposure of the studied items in Session 1 on the hit and false-alarm rates in the indirect recognition test, and for this purpose these dependent variables are analyzed separately (Nishiyama & Kawaguchi, 2014).

Figure 1 shows the structure of the indirect recognition procedure and lists the object sets used for each task in the present experiment. This procedure examines the indirect effect of the incidental learning task in Session 1 on recognition performance in the indirect recognition test of Session 2. The labels “studied” and “nonstudied” mean that the stimuli sets were and were not studied, respectively, in the incidental learning task of Session 1. The label “target” means that the stimuli sets were presented as target objects in the indirect recognition test of Session 2, and that of “distractor” means that the stimuli sets were presented as a distractor in that test. Thus, “studied targets” indicates that the objects were studied in Session 1 and, subsequently, appeared as targets in the indirect recognition test in Session 2. “Nonstudied targets” indicates that the objects were not studied in Session 1 and appeared as targets in the indirect recognition test in Session 2. “Studied distractors” indicates that the objects were studied in Session 1 and appeared as distractor in the indirect recognition test in Session 2. “Nonstudied distractors” indicates that the objects were not presented in any incidental learning task and then appeared as a distractor in the indirect recognition test in Session 2. The numbers in parentheses represent the number of repetitions of each object in each task. Note that “studied targets” and “studied distractors” are both used to examine the effects of incidental learning in Session 1, but they have different roles. In this procedure, Session 2 is regarded as a normal recognition task because it contains both a study task and a recognition test. The purpose of this procedure is to examine the implicit effect of prior experience (i.e., Session 1) that is not directly related to the test on recognition judgment in Session 2. By setting Session 2 as a normal recognition task, “studied targets” are presented not only in Session 1 but also in the incidental learning task of Session 2. Thus, participants’ responses to “studied targets” on the indirect recognition test include not only the effects of incidental learning in Session 2 but also that of Session 1. In contrast, since “studied distractors” do not appear in the incidental learning task in Session 2, participants’ responses to “studied distractors” on the indirect recognition test reflect only the effects of exposure in Session 1. As assuming in this study, if even only slight exposure with objects retained detailed representation for those objects and they effect recognition judgments, then we need to consider the difference in the total number of presentations of “studied targets” and “studied distractors.” That is, it is necessary to consider the possibility that the effects of incidental learning in Session 1 may appear differently for “studied targets” and “studied distractors.” For these reasons, this procedure uses two different subsets of stimuli in Session 1, and the responses to each are compared with those for objects in each nonstudied condition analyzing the hit and false-alarm rates separately. Note that this experimental procedure might be similar to Jacoby’s exclusion/inclusion paradigm (Jacoby, 1991). Jacoby’s paradigm is a method for separating intentional and automatic processes of memory in an explicit memory test, and is similar to the experimental procedure in this study in some ways. For example, both experimental paradigms use a memory test designed to measure explicit memory. In addition, both agree on the theoretical background that performances of explicit memory tests reflect not only intentional recall processes but also automatic processes in participants. However, there are differences between the experimental paradigm used in this study and that used by Jacoby in the following points. First, while the purpose of Jacoby’s exclusion/inclusion paradigm is to separate intentional and automatic processes, the purpose of the indirect recognition procedure used in this study is to detect the long-term persistence of automatic memory processing. Second, Jacoby’s paradigm and our experimental paradigm are similar in that they both focus on automatic memory processes; however, our approach differs from Jacoby’s in that we insert a long interval between the study task and the memory test in order to detect automatic long-term memory.

**Fig. 2:**
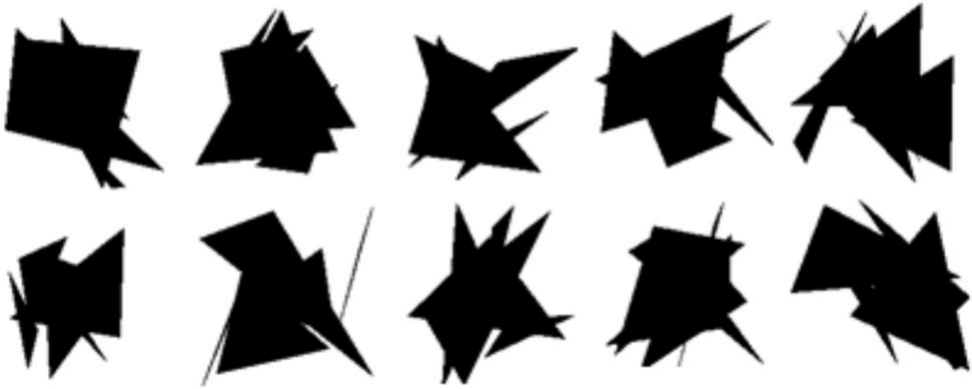
Samples of objects presented in the present experiment

As mentioned above, Session 2 is regarded as a normal recognition task. The prior experience in Session 1 is considered unrelated to the indirect recognition test in Session 2 because participants are only asked to recall the incidental learning task of Session 2. However, implicit effects of the exposure in Session 1 are observed in recognition performance in Session 2. For example, Terasawa and Ohta (1993), using words as stimuli, reported that a prior exposure of stimuli (i.e., Session 1) increased the false-alarm rates in the indirect recognition test about 4 months later (i.e., Session 2). Ueda and Terasawa (2008) also used this procedure, and demonstrated that, even after 14 weeks, auditory representations of random tone sequences formed through incidental learning in Session 1, significantly increased both the hit and false-alarm rates of these representations compared with nonstudied item in the indirect recognition test in Session 2. For nonverbal visual stimuli, our previous studies used objects lacking in semantic information (Fig. 2). We found that representations of objects formed in prior incidental learning are maintained and automatically affect both the hit and false-alarm rate (Nishiyama & Kawaguchi, 2014) or the hit rate alone (Masuoka et al., 2018b).

McKeown et al. (2020) suggested the possibility that representations for sensory visual information may affect a subsequent recognition judgment over long durations. The results of our previous studies using the indirect recognition procedure correspond with this statement; however, these studies did not clarify whether such memory representations are preserved over an extended duration, because there was no interval between study and memory test, and, therefore, only examined whether these representations were retained for a few minutes at longest (Masuoka et al., 2018b; Nishiyama & Kawaguchi, 2014). Thus, in the present study, we used the indirect recognition procedure, inserting a 3-week interval between Session 1 and Session 2, and examined the long-term effects of exposure of the objects which have poor conceptual information on recognition judgments (Fig. 2).

## The present experiment

In addition to examining the long-term persistence of nonverbal objects, the present experiment focused on whether different perceptual operations between study and test affect recognition performance. In a study on the repetition effect for memory, Fendrich et al. (1991) reported that the repetition of perceptual operations facilitated improved recognition performance. Their experiment used a study session in which all participants were presented with three-digit numbers and were asked to type them. After 1 month, a recognition test was carried out using the digits that had been presented in the study session and novel digits. The participants were divided into two groups. The first group was asked to first type the presented digits and then to identify if it was an old or a new item. The other group was asked to first recognize the presented digits and then to type them. In both groups, the exposure frequency of stimuli during the study session was equal. Thus, if a difference in recognition performance for the studied digit lists was observed between the two groups, this would be based on repeating the perceptual operation from the study session (i.e., whether typing the presented digit numbers was performed before recognition judgment or not). They found that the performance of participants who made recognition decisions after typing was better than that of participants who made recognition decisions before typing (Fendrich et al., 1991, Experiment 1). They pointed out that one possible explanation for this effect is that by requiring the participants to type the presented digit before recognition, participants were forced to view the sequence of the digit in the same temporal order as during the study session, and this perceptual overlap between study and test improved recognition performance (Fendrich et al., 1991).

In the present experiment, for the incidental learning task in both sessions, objects were presented individually on a display; participants were asked to count the number of corners of the presented object in a clockwise or anticlockwise direction, based on each participant’s preference, and maintain this direction throughout both sessions. Fendrich et al. (1991) reported that viewing a presented object in a certain direction may involve the acquisition of information about the object in a temporal order. It is possible that when information about an object is acquired in a temporal order, and this temporal order is repeated, this may affect recognition performance. Thus, in the present experiment, we examined whether differences in recognition performance were observed depending on whether the perceptual operation for a presented object was matched between the incidental learning task and the indirect recognition test. Specifically, participants were divided into two groups, and both participated in the incidental learning task of Session 1 and Session 2 along the same manner. In the indirect recognition test, one of the groups was instructed to first count the number of corners of the presented object same as in the incidental learning task, and then to make a recognition judgment. The other group was instructed to make a recognition judgment only. Therefore, only the group that was asked to perform the same operation as in the incidental learning task before making a recognition judgment has the perceptual operation of the presented object matched between study and test.

In summary, the present experiment examined the effects of two factors on recognition performance: one of them was the within-group factor, “object type (nonstudied / studied)” meaning whether the object was studied in the incidental learning task of Session 1, and the other was the between-groups factor, “recognition type (recognition with operation / recognition only),” meaning whether in the indirect recognition test participants performed the same operation as in the incidental learning task. If the representations acquired in the incidental learning task of Session 1 are retained after 3 weeks, there should be differences in recognition performance between the studied object and the nonstudied object. In addition, if the repetition of the perceptual operation plays an important role for recognition judgment based on the representations formed in Session 1, a clear difference related to recognition performance should be observed in the group of participants who were asked to perform the same operation as in the incidental learning task before making a recognition judgment, compared with those in the other group.

## Method

### Participants

A power analysis with G*Power (Faul et al., 2007) suggested that 54 participants were needed to detect a medium effect size (*f* = 0.25) with a power of 0.95 and α = 0.05, for the interaction effect. With reference to this result, we recruited a larger number of participants to account for the possibility that some participants might withdraw from participation in the experiment, considering that this experiment would take three weeks to complete. Thus, we recruited 79 undergraduate students (28 men, 51 women) from Okayama University for this experiment, and they received course credit for participation. They all reported normal or corrected-to-normal vision. Data from 15 participants was excluded from the analysis due to them withdrawing from this study during the experiment or due to experimental errors. Finally, the data of 23 men and 41 women participants were analyzed. Out of the total 64 participants, 32 participants—12 men and 20 women—were in the “recognition with operation” group, and 32 participants—10 men and 22 women participants—were in the “recognition only” group. This assignment was random.

### Apparatus

Object images were presented at a resolution of 1,280 × 1,624 on a 19-in. IPS monitor (EIZO FlexScan S1933) at a refresh rate of 60 Hz. Stimuli presentation and response collection were controlled by E-Prime software (Version 2.0) on a Windows computer. The distance between the participants and the display was approximately 57 cm.

### Stimuli

Stimuli were objects that were created by Nishiyama and Kawaguchi (2014) by randomly combining five black triangles of different sizes and shapes (Fig. 1). Each object subtended a visual angle of four degrees vertically and four degrees horizontally. Nishiyama and Kawaguchi (2014) created 100 objects and surveyed the degree of meaningfulness of each object. Based on the survey, we chose the 40 least meaningful objects. The 40 objects were randomly divided into four stimuli sets: “studied targets,” “nonstudied targets,” “studied distractors,” and “nonstudied distractors,” with 10 objects each which were used in the main experimental trials. These stimuli sets were counterbalanced across participants. In addition, to reduce primacy and recency effects, six objects were used as filler items—three of them were inserted at the beginning and the other three at the end of all tasks. Filler items were not included in the 40 objects used in the main experimental trials, and responses to them were excluded from analyses.

### Procedure

**Fig. 3:**
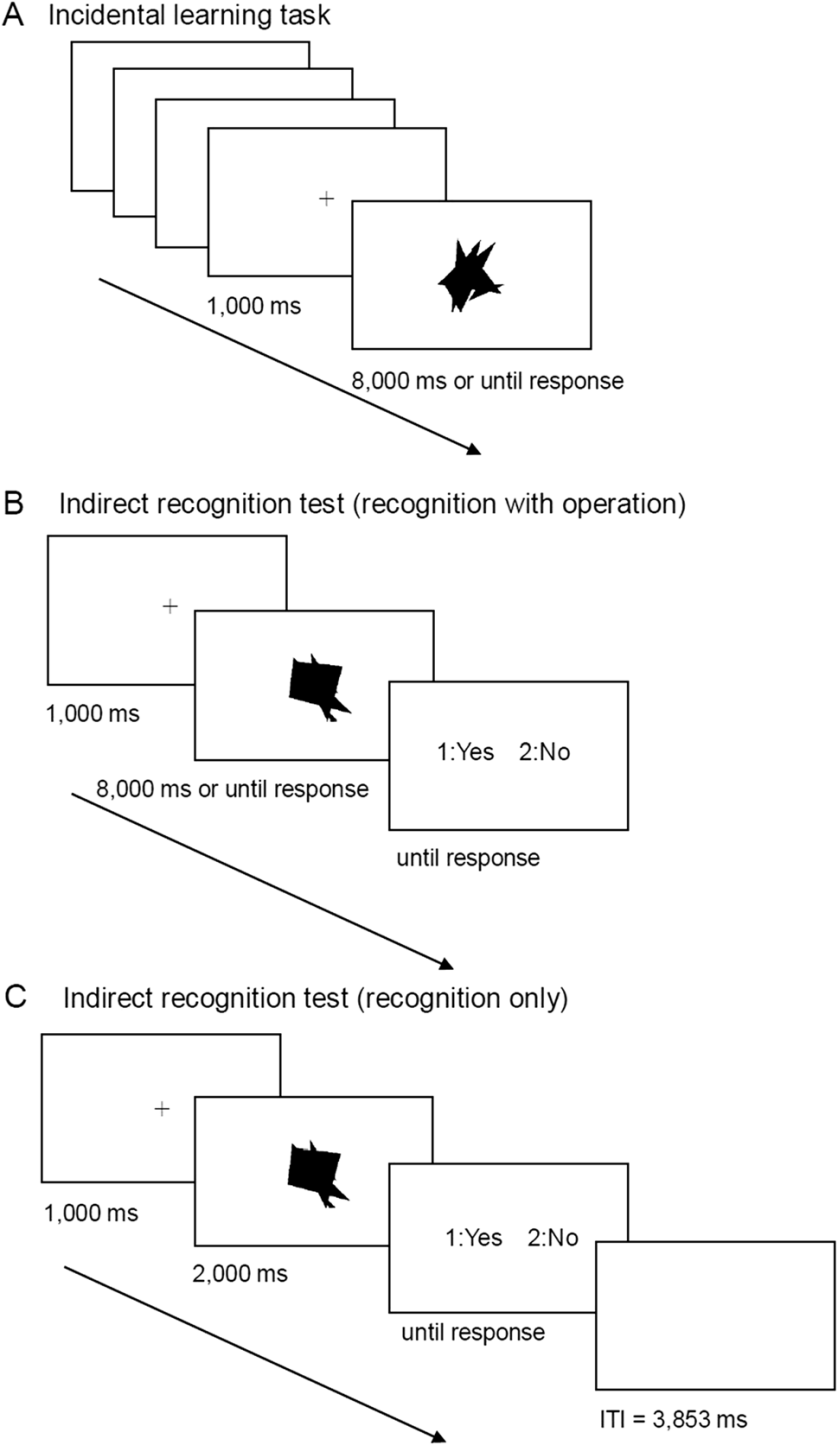
Schematic incidental learning task (**A**), the indirect recognition test for the “recognition with operation” group (**B**), and the indirect recognition test for the “recognition only” group (**C**) in the experiment

This experiment consisted of two sessions. Figure 3 shows the procedure, and Fig. 1 shows the list of object sets used for each task in the experiment. Session 1 was an incidental learning task (Fig. 3A). A black fixation cross was presented, and the experimenter pressed the enter key to initiate the trial. Objects were presented individually on the display and participants were asked to count the number of corners of the presented object and press the appropriate number key while the object was being presented. The object was presented on the display until a response key was pressed or until 8,000 ms passed. In addition, the participants were instructed to count in a clockwise or anticlockwise direction based on their preference when counting the number of corners of the presented object and to use the same counting method for all the objects presented. In this task, the two stimuli sets were used: “studied targets” and “studied distractors.” These objects were presented twice, resulting in 40 trials, without counting the practice and filler trials. The presentation order was random, except for the adjustments made to ensure that the same stimuli would not be continuously presented. Two practice trials were conducted before the experimental trials.

In Session 2, which was conducted after 3-weeks interval, the participants were asked to perform the same incidental learning task as Session 1. In this task, when counting the number of corners of the presented objects, the participants were instructed not only to unify the counting direction within this task but also to use the same direction as the one used in Session 1. This aimed to unify the view for the presented object with Session 1. Except for this instruction, the task was carried out in the same manner as Session 1. In this task, the two stimuli sets were used: “studied targets” that had already been presented in Session 1 and “nonstudied targets” that were novel. Each object was randomly presented once.

Immediately afterwards, the indirect recognition test was performed. In this test, different procedures were used for each group. For the “recognition with operation group” (Fig. 3B), a black fixation cross was centered on the display for 1,000 ms, and each object was individually displayed. The participants were asked to count the number of corners of the presented object and press the appropriate number key, the same procedure as the incidental learning task. The object was presented on the display until a response key was pressed or until 8,000 ms passed. The participants were then asked to identify whether the presented object had appeared in the incidental learning task of Session 2 and to press the appropriate key (1: Yes, 2: No). The test display was presented on the screen until the participants responded. For the “recognition only” group (Fig. 3C), each object was individually presented for 2,000 ms. Participants were asked to identify whether the presented object had appeared in the incidental learning task of Session 2 and to press the appropriate key (1: Yes, 2: No). There was an intertrial interval (ITI) of 3,853 ms immediately after each recognition response. This ITI was inserted to unify the overall trial time of the “recognition with operation” group and reduce primacy and recency effects. The length of the ITI was determined based on the average response time of the incidental learning task in the prior experiment of Masuoka et al. (2018a), which conducted the same experimental procedure as the present study. In this test, for both of two groups, all the four stimuli sets were presented: “studied targets,” “nonstudied targets,” “studied distractors,” and “nonstudied distractors.” These objects were each randomly presented once.

## Results

**Fig. 4:**
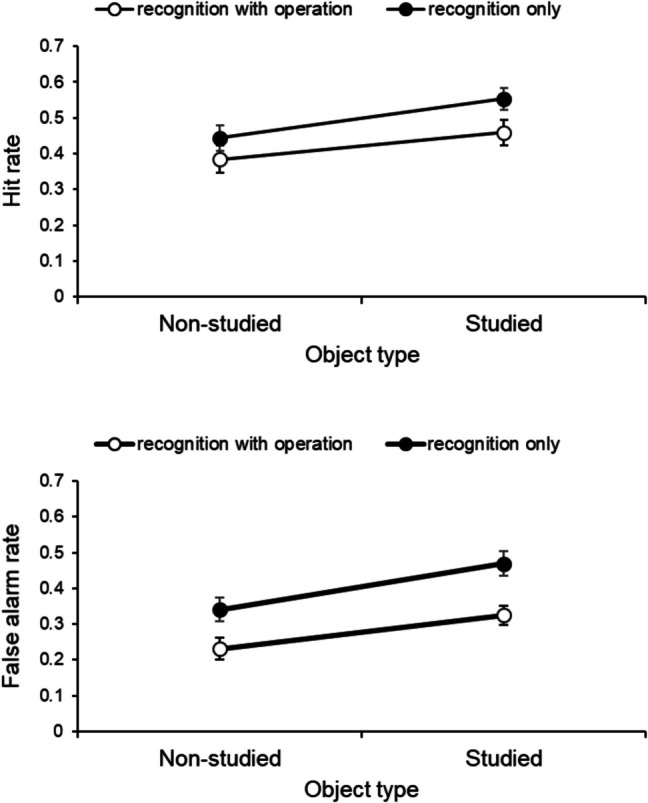
Mean hit and false-alarm rate as object type for each group of recognition type. Error bars indicate standard errors

The length of the interval between Sessions 1 and 2 varied between participants, with an average of 21.19 days (*SD* = 0.47 days). When the participants performed the indirect recognition test, they were asked to identify whether the presented object had appeared in the incidental learning task of Session 2. Thus, for each of the “studied targets” and “nonstudied targets,” the rates of “Yes” responses were calculated as the hit rates. For both the “studied distractors” and “nonstudied distractors,” the rates of “Yes” responses were calculated as the false-alarm rates. Figure 4 shows the average hit and false-alarm rates for each group. For each hit and false-alarm rate, we used a 2 (object type: nonstudied, studied) × 2 (recognition type: recognition with operation, recognition only) analysis of variance (ANOVA). Object type was the within-group factor and recognition type was the between-groups factor. All analyses were performed using the statistical software JASP (Version 0.16.1; JASP Team, 2022).

### Hit rate

The ANOVA revealed the main effect of object type, *F*(1, 62) = 13.34, *p* = 0.00, η_p_^2^ = 0.18, indicating that the hit rate for “studied targets” was significantly higher than that for “nonstudied targets.” There was no significant difference in the main effect of the recognition type, *F*(1, 62) = 3.07, *p* = 0.09, η_p_^2^ = 0.05, or the object type and recognition type interaction, *F*(1, 62) = 0.46, *p* = 0.50, η_p_^2^ = 0.01.

### False-alarm rate

The ANOVA revealed the main effect of object type, *F*(1, 62) = 21.96, *p* = 0.00, η_p_^2^ = 0.26, indicating that the false-alarm rate for “studied distractors” was significantly higher than that for “nonstudied distractors.” The main effect of recognition type was also confirmed, *F*(1, 62) = 11.67, *p* = 0.00, η_p_^2^ = 0.16, indicating that the false-alarm rates, when the operations at the incidental learning task and the indirect recognition test are matched, was significantly lower than that for when the operations performed at the incidental learning task was not required at the indirect recognition test. There was no object type and recognition type interaction, *F*(1, 62) = 0.53, *p* = 0.47, η_p_^2^ = 0.01.

### *d*′

As previously stated, the purpose of this study was not to examine whether our participants could correctly discriminate between target and distractor objects in the indirect recognition task, but to examine the implicit effect of prior experience that was not directly related to the test on recognition judgment. For this purpose, in addition to Session 2, which is considered a normal recognition task, we have inserted Session 1 as an incidental learning task, which is performed earlier. Because the effects of incidental learning in Session 1 may have differed between “studied targets” and “studied distractors” due to differences in the number of presentations, we analyzed the hit and false-alarm rates separately.

However, we additionally calculated *d′* as a measure of the participants’ performance in the indirect recognition test for the following two reasons. First, the hit and false-alarm rates still include the participants’ response bias. Second, because many of the studies for visual long-term memory have reported *d*’ as a performance of memory task, it is necessary to provide a quantitative indicator to compare and discuss the results of those studies and the present study.

**Fig. 5:**
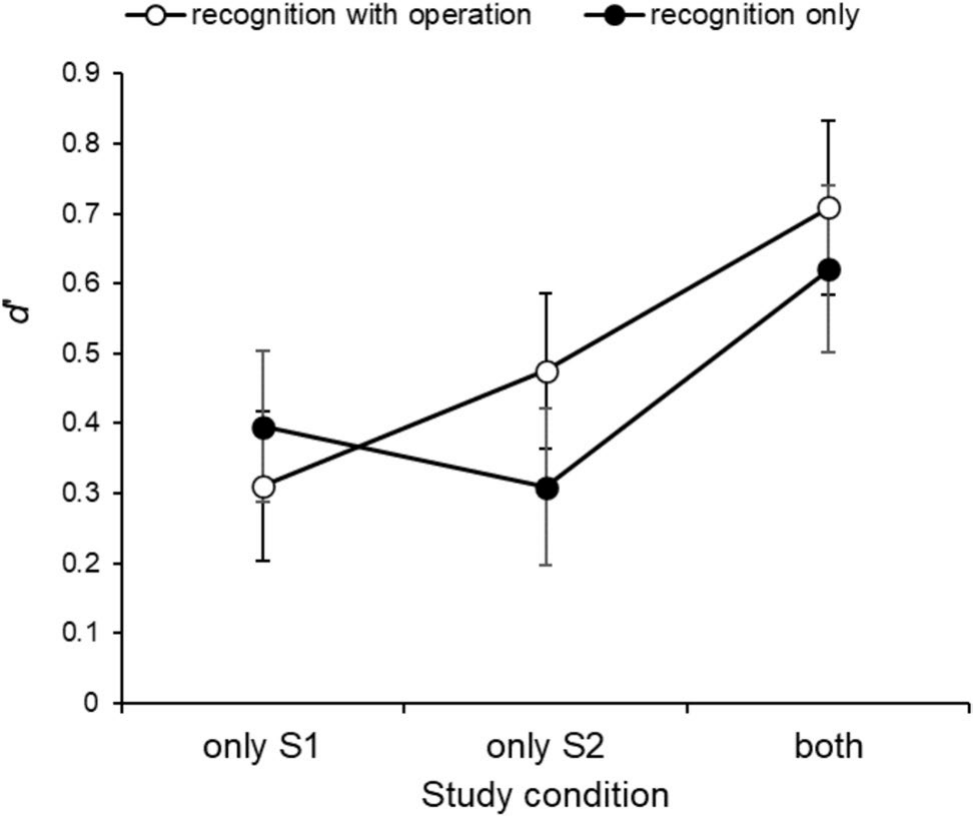
Mean *d*′ as the study condition for each group of recognition type. Error bars indicate standard errors

Because the indirect recognition procedure we used in this study differ from the usual recognition task, there were two types of distractors presented in the recognition test in Session2: “studied distractors” and “nonstudied distractors.” Of these, “studied distractors” were items that have already been studied in Session1, so that only responses to “nonstudied distractors” provide the genuine noise distribution. Therefore, we calculated three types of *d′* as follows: The first of the three is the value of *d′* that is based on the responses to “studied distractors” presented only in Session1 and the responses to “nonstudied distractors,” and we called this condition “Only S1.” Second is the value of *d′* that is based on the responses to “nonstudied targets” presented only in Session 2 and the responses to “nonstudied distractors,” and we called this condition “Only S2.” The third one is based on the responses to “studied targets” presented in both Sessions and the responses to “nonstudied distractors,” and we called this condition “both.” Fig. 5 shows the average *d′* for each condition. For the analysis of *d′*, we used a 3 (study condition: Only S1, Only S2, and both) × 2 (recognition type: recognition with operation, recognition only) ANOVA. The main effect of study condition was confirmed, *F*(2, 124) = 11.04, *p* < 0.001, η_p_^2^ = 0.15. As a result of multiple comparisons using the Holm method, the *d′* for “both” condition was significantly higher than the *d′* for the other two conditions (vs. “Only 1”, *p* < 0.001; vs. “Only 2”, *p* < 0.001). There was no main effect of the recognition type, *F*(1, 62) = 0.17, *p* = 0.68, η_p_^2^ = 0.003, or the interaction, *F*(2, 124) = 1.57, *p* = 0.21, η_p_^2^ = 0.025. Since the main effect of recognition type was not observed, we ignored a distinction by recognition type, and confirmed that all three *d′* values were significantly greater than 0—for “Only 1”, *t*(63) = 4.68, *p* < 0.001, *d* = 0.59; for “Only 2”, *t*(63) = 4.97, *p* < 0.001, *d* = 0.62; for “both” *t*(63) = 7.74, *p* < 0.001, *d* = 0.97.

Finally, we investigated the differences in the bias between participants’ groups. As a result, the criteria for judgments of the “recognition with operation” group were significantly higher than that of the “recognition only” group, *t*(190) = 4.74, *p* < 0.001, *d* = 0.15.

## Discussion

### Visual long-term memory for nonverbal objects

The major purpose of the present study was to examine the effect of prior experiences that were difficult to consciously recall on subsequent recognition judgments, using novel objects and longer delay. Both the hit and false-alarm rates for the studied objects in the indirect recognition test were significantly higher than those for the nonstudied objects, indicating that the representations of nonverbal objects formed in Session 1 were maintained for a long duration, approximately 3 weeks. The results for *d*’ also led to the same conclusion. The performance of discrimination for the “both” condition was significantly better than that for the other two conditions, indicating the long-term effect of prior experience on a subsequent recognition judgment. Recent studies for visual long-term memory demonstrated that much more detailed representations of daily objects and scenes are retained in memory than we are conscious of (e.g., Brady et al., 2008; Hollingworth, 2005; Konkle et al., 2010a, 2010b; Miner et al., 2020). Konkle et al. (2010b) showed that conceptual distinctiveness predicts high memory performance, and that preexisting conceptual knowledge of objects supports detailed representations in long-term memory. Although the present study did not directly examine the effects of the conceptual distinctiveness of objects, it seems likely that there was low conceptual discriminability between the studied and nonstudied objects, and that both sets were novel to the participants. Nevertheless, the observed difference in the recognition performance between the studied and nonstudied objects implies that the representations formed in Session 1 were preserved precisely, to the extent of causing a difference in the recognition judgment, even without preexisting conceptual knowledge. Previous studies of priming for nonverbal visual information have reported that a brief exposure to stimuli is enough to form detailed memory representations (DeSchepper & Treisman, 1996; Musen & Treisman, 1990), and the results of the present study support this claim.

Note that, however, it is not possible with the present data to make a strong determination on whether the stimuli used in this study were truly meaningless for the participants and that there was absolutely no elaborative encoding process. Brady et al. (2019) reported that there were individual differences in whether the object is perceived as meaningful or not. Even if the objects were lacking in conceptual information, memory representations corresponding to conceptual information and connected to preexisting knowledge might have been created by exposure in Session 1. Thus, further research is needed to examine what information is encoded in memory when we are exposure with visual information.

### Effects of matching of perceptual operation on recognition

Another purpose of this study was to examine whether repeating the same perceptual operation used in the incidental learning task affected recognition performance. The result showed that the effect of performing the same operation as the incidental learning task before making recognition judgment was confirmed only in false-alarm rates. We calculated the criteria for recognition judgment for each group and compared them. As a result, we found that the criteria for the “recognition with operation” group were stricter than those for the “recognition only” group. Considering that no main effect or interactions of the recognition type were observed in the hit rates and *d′* and that there were differences between the recognition type in the judgment criteria, the overall lower the hit and false-alarm rates in the “recognition with operation” group can be interpreted as indicating that the participants were simply more prone to reject objects under this condition, rather than that the nature of memory changed due to performing the same operation as the incidental learning task before making judgment. Fendrich et al. (1991) reported that instructing participants to type a digit in the study session and doing the same typing operation before recognition in the memory test, benefits recognition performance. They explained that matching operations in the study and test sessions forced participants, when making a recognition judgment, to view the digit list in the same temporal order as they had viewed it in the study session and that this perceptual overlap may provide an advantage in recognition performance. In the present study, for the participants of both groups, there is a possibility that information of temporal order for the presented objects was encoded through the operation of counting the number of corners of the objects in the incidental learning task. However, only the “recognition with operation” group was asked to view the objects on the same temporal order in the indirect recognition test as they had in the incidental learning task. Notably, the results of the present study were not consistent with those of Fendrich et al. (1991) and the effect of matching perceptual operation is unclear in the present data. Thus, further research is needed to examine this effect, and to verify specific information that is contained in maintained representations for nonverbal visual information.

### Limitations and conclusion

Although the results of the present study show that representations of nonverbal visual objects are retained for a long duration, this paper only reports the phenomenon and cannot state the specifics of its functioning or the specific processes that improved recognition performance. The present study is built on previous studies of nonverbal visual information that showed the effects of prior exposure that is not directly related to the episode that participants were required to recall on a recognition test (Masuoka et al., 2018b; McKeown et al., 2014, 2020; Nishiyama & Kawaguchi, 2014), and we detected its long-term effects on recognition performance. In the present experiment, participants were not asked to recall the episode of Session 1 when they performed the indirect recognition test, and so the effects of exposure of the objects in Session 1 observed in the recognition performance seem to be formally based on implicit memory for the “Studied targets” and the “Studied distractor.” However, we cannot empirically rule out the possibility that the participants might have tried to memorize the presented objects intentionally in Session 1 or that they recalled the episode of Session 1 when performing the indirect recognition test. Thus, we cannot clearly distinguish whether the effects of exposure to the objects in Session 1 on the recognition performance were based on implicit or explicit memory. However, considering that the objects were difficult to encode verbally, and the participants were not asked to intentionally memorize the objects in the incidental learning task in Session 1, it would have been difficult for them to consciously maintain the representations of each object presented in Session 1 until the indirect recognition test 3 weeks later. Thus, the effects of exposure in Session 1 on the recognition performance observed in the present experiment seems to reflect an automatic process.

McKeown et al., (2014, 2020) indicated that representations for sensory visual information are difficult to maintain intentionally but are maintained automatically, demonstrating that exposure to stimuli in a prior trial affected subsequent recognition performance in unrelated tests. Although the experimental procedure in the present study was different from that of McKeown et al., (2014, 2020), the results of the present study are consistent in that we detected the effects of brief, unrelated prior exposure on recognition performance. Additionally, the present study demonstrated that the effects persisted for a long duration. Recognition tasks were originally used as a procedure to detect consciously recalled memory; however, the results of the present study suggest that this procedure can be used to detect an automatic effect of even the briefest experience on memory. That is, each specific prior experience of a visual object is retained in long-term memory and affects memory performance (Terasawa, 2005). The results of the present study suggest that the effect of even the briefest prior experience on recognition judgment should not be underestimated. This might also be inferred, for example, from reports that detailed visual long-term memory for everyday objects are formed and retained even in an incidental learning situation (Castelhano & Henderson, 2005; Williams et al., 2005), and from findings that prior experiences in everyday life that are not directly related to a memory test in an experiment enhance memory test performance (Miner et al., 2020, Experiment 4). Further research is needed to clarify how specific experiences are represented independently in memory and the specific processes involved in how these representations affect memory performance.

## Data Availability

The data for the experiment reported here and the material can be obtained by contacting the corresponding author. The experiment was not preregistered.
